# A Randomized, Double-Blind, Placebo-Controlled Trial: The Efficacy of Multispecies Probiotic Supplementation in Alleviating Symptoms of Irritable Bowel Syndrome Associated with Constipation 

**DOI:** 10.1155/2016/4740907

**Published:** 2016-08-09

**Authors:** Valerio Mezzasalma, Enrico Manfrini, Emanuele Ferri, Anna Sandionigi, Barbara La Ferla, Irene Schiano, Angela Michelotti, Vincenzo Nobile, Massimo Labra, Patrizia Di Gennaro

**Affiliations:** ^1^Department of Biotechnology and Biosciences, University of Milano-Bicocca, Piazza della Scienza 2, 20126 Milan, Italy; ^2^Farcoderm Srl., Via Angelini 21, San Martino Siccomario, 27028 Pavia, Italy

## Abstract

*Background and Aim*. The efficacy of supplementation treatment with two multispecies probiotic formulates on subjects diagnosed with IBS-C and the assessment of their gut microbiota were investigated.* Methods*. A randomized, double-blind, three-arm parallel group trial was carried out on 150 IBS-C subjects divided into three groups (F_1, F_2, and F_3). Each group received a daily oral administration of probiotic mixtures (for 60 days) F_1 or F_2 or placebo F_3, respectively. Fecal microbiological analyses were performed by species-specific qPCR to assess the different amount of probiotics.* Results*. The percentage of responders for each symptom was higher in the probiotic groups when compared to placebo group during the treatment period (*t*60) and was maintained quite similar during the follow-up period (*t*90). Fecal analysis demonstrated that probiotics of the formulations increased during the times of treatment only in fecal DNA from subjects treated with F_1 and F_2 and not with F_3, and the same level was maintained during the follow-up period.* Conclusions*. Multispecies probiotic supplementations are effective in IBS-C subjects and induce a different assessment in the composition of intestinal microbiota. This clinical study is registered with the clinical study registration number ISRCTN15032219.

## 1. Introduction

The intestinal microbiota consists of a wide range of bacterial species [1]. The microbial community resides in the gut of the host establishing a mutually beneficial relationship and modulating, through its metabolic activities, the host's health status [2, 3]. The microbiota exerts different physiological functions such as inhibition of pathogenic bacteria and synthesis of short fatty acids; stimulation of nutrient and mineral absorption, as well as modulation of the intestinal immune system; synthesis of vitamins and amino acids; and the decomposition of protein compounds [4, 5]. The intestinal microbiota in healthy adults is generally considered stable over time in predominance of bacteria belonging to four main phyla: Bacteroidetes and Firmicutes as prevalent and Actinobacteria and Proteobacteria that are less represented. The microbiota in subjects with Irritable Bowel Syndrome (IBS) has been shown to be less stable compared to healthy adults [5, 6]. A controlled balance between bacterial species considered beneficial (lactobacilli and bifidobacteria) associated with the reduction of those considered harmful (*Clostridium*,* E. coli*,* Salmonella*, and* Pseudomonas*) is fundamental for maintenance of the gut physiological state and should attenuate IBS symptoms. Alteration of the microbiota's composition, which can be caused by psychophysical, dietary, or environmental stress, is defined as dysbiosis and can lead to pathological conditions. Moreover, aberrant stimulation of the immune system leading to inflammation may be reliably encountered as the potential link between dysbiosis and metabolic diseases [7–9].

Experimental evidences have increasingly shown a correlation between microbiota imbalance and the induction of Irritable Bowel Syndrome. In particular, dysbiosis was proven to underlie a modification of intercellular tight junctions, responsible under normal conditions for the correct structure of the epithelial layer of intestinal mucosa, which is known to increase the mucosal permeability. Consequently, antigens have been detected in the intercellular space with the activation of the inflammatory cascade, production of cytokines, and tissue damage [9–11].

Oral administration of appropriate probiotic strains may therefore be beneficial to health and in particular with respect to IBS. However, clinical data reporting changes in the detection of ingested probiotic traceability in the gastrointestinal tract of patients and the related effects on the gut are still few and inconclusive [12–15]. Studies have reported an improvement in global symptoms with probiotics, while others have failed to demonstrate any benefit [16–18].

Moreover, it is widely known that the effect of probiotics is species-specific [19, 20]. These indications combined with the diversity and complexity of IBS may indicate that a probiotic combination could be more efficient than a single strain in this particular syndrome. Some authors suggested that multispecies probiotics may in some conditions be more efficient than monospecies probiotics due to, for example, enhanced intestinal adhesion and the production of a greater variety of antimicrobial compounds, compared with single probiotic [15].

In this regard, the present study was aimed at testing different probiotics, which we had previously characterized [19] for their* in vitro* activities, in order to demonstrate by means of* in vivo* detection procedures their distinctive effects on the intestine after oral administration. The aim of this randomized double-blind, placebo-controlled study was to investigate the efficacy of two probiotic formulations in ameliorating IBS-C subjects. The effects of probiotics on IBS symptoms in comparison with placebo and their assessment in the gut microbiota after probiotic therapy by analysing fecal bacteria were evaluated. In order to reach this goal, 150 volunteer subjects were recruited and included in three groups (F_1, F_2, and F_3). Each group received 60-day oral administration of different probiotic mixtures: F_1 containing* L. acidophilus* and* L. reuteri*; F_2 containing* L. plantarum*,* L. rhamnosus*, and* B. animalis *subsp.* lactis*; and F_3 containing placebo. At different time points after probiotic oral administration, fecal microflora was analysed by real-time quantitative PCR performed on DNA from stool samples of the subjects. An additional 30-day follow-up period was encountered in order to assess whether the observed beneficial effects still persist after treatment suspension.

Our investigation provides a relevant contribution to investigate the efficacy of multispecies probiotics in treating IBS-C and other gastrointestinal syndromes related to the gut disorders.

## 2. Materials and Methods

### 2.1. Study Design

This randomized, double-blind study was carried out in accordance with the Declaration of Helsinki and the Good Clinical Practice guidelines E6. The study protocol and the informed consent form were approved by the “Independent Ethical Committee for Non-Pharmacological Clinical studies” during its meeting on July 17, 2013. All subjects provided a written informed consent before initiation of any study-related procedures. The study took place at Farcoderm Srl. facilities. Farcoderm Srl. is an independent testing laboratory for* in vitro* and* in vivo* safety and efficacy assessment of cosmetics, food supplements, and medical devices.

Enrolled subjects were randomly assigned to receive one capsule of the two different formulations of probiotics (mix F_1 and mix F_2) or placebo (mix F_3) once daily for a period of 60 days and were followed up for a further period of 30 days after a follow-up period from the last ingestion of the tested products.

The tested products consisted of food supplements (capsules) containing lactobacilli and bifidobacteria (Principium Europe Srl., Solaro, MI, Italy) (Table 1). The composition of the probiotic mix F_1 was as follows: 5 × 10^9^ CFU* L. acidophilus* (30 mg as lyophilized), 5 × 10^9^ CFU* L. reuteri *(30 mg as lyophilized), 330 mg inulin, 5 mg silica, and 5 mg talc. The F_2 composition was as follows: 5 × 10^9^ CFU* L. plantarum* (12 mg as lyophilized), 5 × 10^9^ CFU* L. rhamnosus *(20 mg as lyophilized), 5 × 10^9^ CFU* B. animalis *subsp*. lactis *(60 mg as lyophilized), 298 mg inulin, 5 mg silica, and 5 mg talc. Placebo (F_3) composition was as follows: 390 mg inulin, 5 mg silica, 5 mg talc.

**Figure 1:**
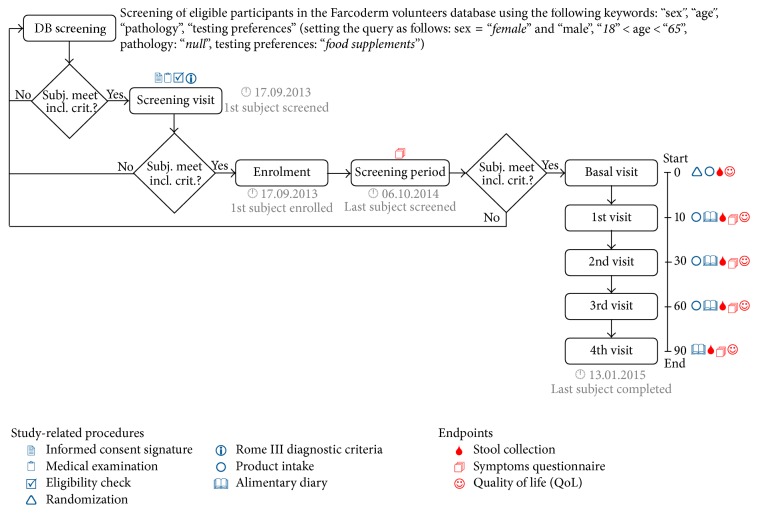
Study flow and schedule of assessment chart.

The study flow and the schedule of assessment chart is reported in Figure 1. A questionnaire and an explanation of the protocol of the study were given to the subjects. Symptom questionnaire was performed as an interview to the enrolled subjects at the time points of the study (each 10 days).

Stool samples for fecal microbiota analysis were obtained at the start of the treatment (*t*0) and at times *t*10, *t*30, and *t*60 days during the treatment for a total period of 60 days, followed by a sample at *t*90 days after washing up for 30 days, for a total period of 90 days from the start of the treatment.

### 2.2. Subjects of the Study

Eligible subjects were all adult males and females aged between 18 and 65 years suffering from Irritable Bowel Syndrome with constipation (IBS-C) diagnosed by clinical analyses and self-reported interviews. Subjects suffering from IBS-C were screened by means of the Rome III diagnostic criteria questionnaire [21]. Exclusion criteria were (i) pregnancy or the intention to become pregnant, (ii) lactation, (iii) food intolerances/allergy, (iv) known history of other gastrointestinal disorders, (v) chronic or acute gastrointestinal disorders, (vi) participation in another similar study, and (vii) unwillingness or inability to comply with the study protocol requirements.

The study further excluded subjects using food supplements (included probiotics different from the study) or drugs containing actives having an influence on gastrointestinal physiology. Changes in diet or lifestyle were not allowed during all the study period.

### 2.3. Assessment of Clinical Effects

#### 2.3.1. Endpoints

Patients were evaluated five times during the course of the study: at baseline, after 10, 30, and 60 days from the start period, and 30 days after the follow-up of the intervention period to assess postintervention effects. Primary efficacy endpoints were the proportion of participants whose IBS symptoms after probiotic supplementations were relieved up to 60 days and the assessment of their gut microbiota. The secondary efficacy endpoint was the maintenance of the obtained effects 30 days after the last product(s) intake.

#### 2.3.2. Symptoms Questionnaire

IBS-C related symptoms of subjects were reported daily by a questionnaire according to Guide Lines of FDA (Guidance for Industry-Irritable Bowel Syndrome-Clinical Evaluation of Drugs for Treatment). IBS-C questionnaire consisted of 5 items as follows: (i) bloating, (ii) abdominal pain, (iii) constipation, (iv) abdominal cramps, and (v) flatulence. For each item subjects scored the symptom severity on a 10-point Visual Analogue Scale (VAS). Data are reported as the mean values after every 10 days.

#### 2.3.3. Health-Related Quality of Life (HR-QOL)

HR-QOL was assessed by means of the Italian version of the Quality of Life Measure for Persons with IBS [22]. From the original 34 items questionnaire, the following 12 items were selected: (i) I am embarrassed by the smell caused by my bowel problems, (ii) I feel vulnerable to other illnesses because of my bowel problems, (iii) I feel fat because of my bowel problems, (iv) I feel my life is less enjoyable because of my bowel problems, (v) I feel depressed about my bowel problems, (vi) I have to watch the amount of food I eat because of my bowel problems, (vii) because of my bowel problems, sexual activity is difficult for me, (viii) I feel angry that I have bowel problems, (ix) I feel I get less done because of my bowel problems, (x) my bowel problems limit what I can wear, (xi) I have to watch the kind of food I eat because of my bowel problems, and (xii) I fear that I won't be able to have a bowel movement.

For each item, the following five-point response scale was used: 1: not at all, 2: slightly, 3: moderately, 4: quite a bit, and 5: extremely. HR-QOL was interviewer-administered.

### 2.4. Fecal Samples (Stools Type Classification)

Fecal samples were collected from subjects at *t*10, *t*30, *t*60, and *t*90 time points during the study. Fresh fecal samples were homogenized by vortex mixing of the fecal mass and separated into aliquots to be stored at −80°C until the analysis using DNA-based method quantitative PCR.

Stool types were classified according to Bristol scale [23]. Bristol values were divided in two categories: values 3 (sausage shape with cracks in the surface, normal), 4 (smooth soft sausage or snake, normal), and 5 (soft blobs with clear-cut edges, lacking fiber), corresponding to healthy bowel situation, were assigned to class 1, whereas values 1 (separate hard lumps, very constipated), 2 (lumpy and sausage like, slightly constipated), 6 (mushy consistency with ragged edges, inflammation), and 7 (liquid consistency with no solid pieces, inflammation) were assigned to class 0. The number of volunteer subjects with stool samples with Bristol values belonging to class 1 was calculated for each treatment and for each experimental time.

### 2.5. Probiotic Strains and DNA Extraction

In this study, a total of four* Lactobacillus* spp. strains and one* Bifidobacterium* strain, supplied from a private collection, were taken into consideration for the preparation of the two formulations (F_1 and F_2). Table 1 describes the characteristics of each strain. In order to prepare standard curves of DNA extracted from probiotics, microbial cultures were performed in MRS (Conda) medium and incubated at 37°C for 24 hours in anaerobic conditions using anaerobic atmosphere generation bags (Anaerogen, Oxoid). For* B. animalis *subsp*. lactis*, a supplementation of 0.3 g/L L-cysteine hydrochloride monohydrate was included in the growth medium (cMRS) (Sigma-Aldrich).

DNA from microbial cultures was extracted by the ZR fecal DNA MiniPREP (Zymo Research). A total of 1 mL of 10^9^ CFU/mL culture was used for obtaining genomic DNA following the protocol provided by the manufacturer.

DNA extraction from stool samples was performed from 150 mg of feces by the ZR fecal DNA MiniPREP. Both DNA extracted from probiotic cultures and DNA from stool samples were utilized to perform qPCR.

### 2.6. Fecal Microbiology Analysis by Quantitative PCR

qPCR reactions were carried out using an ABI 7500 (Applied Biosystems) and the SsoFast EvaGreen Supermix with Low ROX (BIO-RAD) dye. We designed species-specific primer sets developed by the authors in a previous study and reported in Table 2 [24]. Reactions were carried out in a 10 *μ*L qPCR mix containing 5 *μ*L of SsoFast EvaGreen Supermix with Low ROX, 0.2 *μ*L of 10 *μ*M forward primer and 10 *μ*M reverse primer, 4 *μ*L of DNA template, and 2.4 *μ*L of H_2_O, according to the following qPCR program: 10′ 95°C and 40 cycles of 15′′ 95°C and 1′ 60°C (followed by a dissociation step).

For each strain, standard curves were constructed using DNA extracted from microbial cultures using tenfold dilutions ranging from 10^8^ CFU/mL to 10 CFU/mL. Each DNA sample both from feces and from culture dilution was analysed in triplicate.

### 2.7. Sample Size

Sample size was calculated with a two-side 5% significance level and a power of 80% taking into account a 23 mm margin of equivalence among treatments. A sample size of 50 subjects per treatment arm was considered necessary given an anticipated dropout rate of 40%. Taking into consideration the duration of the treatment and the complexity of the inclusion, we expected a high rate of dropout.

### 2.8. Randomization

Subjects were assigned to treatment arms using a computer-generated PASS 11 statistical software (version 11.0.8 for Windows; PASS, LLC, Kaysville, UT, USA) restricted randomization list (“Efron's biased coin” algorithm). Subjects were randomized in a 1 : 1 : 1 (F_1 : F_2 : F_3) ratio. The software was running on Windows Server 2008 R2 Standard SP1 64 Edition (Microsoft, USA). Subjects, investigator, and collaborators were kept blind to products assignment. The randomization list was stored in a safe place by the* in site* study director.

### 2.9. Statistical Methods

Statistical analysis was performed using NCSS 8 (version 8.0.4 for Windows; NCCS, LLC) running on Windows Server 2008 R2 64 Edition. Internal consistency was checked before statistical analysis in order to assess subject's reliability. For IBS-C related symptoms, the number of responders to treatment was calculated. A responder was defined as the subject reporting a decrease of symptoms of at least 30% compared to the basal condition for at least 50% of the intervention time (Guidance for Industry-Irritable Bowel Syndrome-Clinical Evaluation of Drugs for Treatment). Positive/negative responses to treatment/placebo were tested using Fisher's exact ratio test. HR-QOL and follow-up data were submitted to RM-ANOVA followed by Tukey-Kramer posttest. Data normality was checked using skewness, kurtosis, and omnibus test. Statistical significance was reported as follows: ^*∗*^ *P* < 0.05, ^*∗∗*^ *P* < 0.01, and ^*∗∗∗*^ *P* < 0.001.

In order to apply generalized linear mixed model (GLMM) under Poisson-lognormal error to account for higher variation at the lower end of target abundance, MCMC.qPCR R package [25] was used to convert Ct data in bacterial counts. The conversion to approximate counts uses the following formula:

(1) $$\begin{array}{c} \text{Count: }E^{\left(Ct1-Ct\right)}, \end{array}$$

where *E* is the efficiency of amplification and Ct1 is the number of qPCR cycles required to detect a single target molecule.

Markov Chain Monte Carlo (MCMC) algorithm implemented in the package is used to sample from the joint posterior distribution over all model parameters, in order to estimate the effects of all experimental factors on the levels of specific microbial species. GLMM was used to test whether the levels of the different microbial species in different formulation groups (F_1, F_2, and F_3) differed between the baseline (*t*0) and the subsequent time points (*t*10, *t*30, *t*60, and *t*90).

The experimental design is incorporated into the following model:

 ln(counts)  ~  species + species:Formulation + species:Time +* sample* +* species:sample* +* species:residual*,

where the logarithm of bacterial counting rate is the variable response and the fixed factors are Formulation and Time (baseline and subsequent time points). The three remaining factors* sample* (different subjects of the study),* species:sample*,and* species*:*residual* are defined as random factors, accounting for the variation in quality and quantity of biological material among samples.

To produce graphical chart, we used ggplot2 R package [26].

## 3. Results

### 3.1. Subjects of the Study

**Figure 2:**
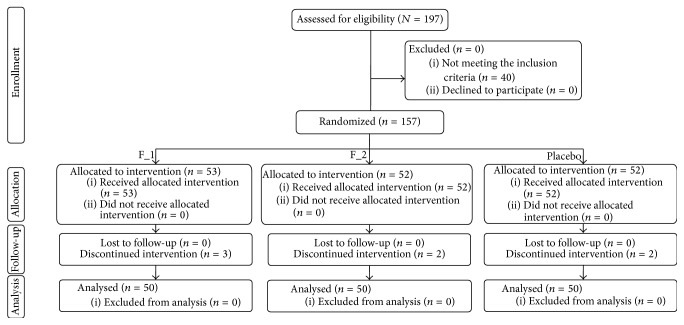
Disposition of the subjects of the study.

The study was conducted between September 2013 and January 2015. A total of 157 male and female subjects suffering from IBS-C were successfully enrolled (Figure 2). Subjects were randomized to active or placebo treatments as follows: (i) 53 subjects were randomized to F_1, (ii) 52 subjects were randomized to F_2, and (iii) 52 subjects were randomized to F_3. Seven subjects discontinued intervention because they were no longer interested in participating in the study.

After randomization, subjects attended four clinic visits every month, except for the first visit (10 days after product use). The population was Caucasian and the mean (±SD) age was 37.4 ± 12.5 years. Demographic and baseline characteristics (Table 3) were similar across treatment arms, indicating an unbiased randomization. The per-protocol population consisted of 150 subjects. All subjects were included in the safety analysis dataset.

### 3.2. Primary Efficacy Endpoint

**Figure 3:**
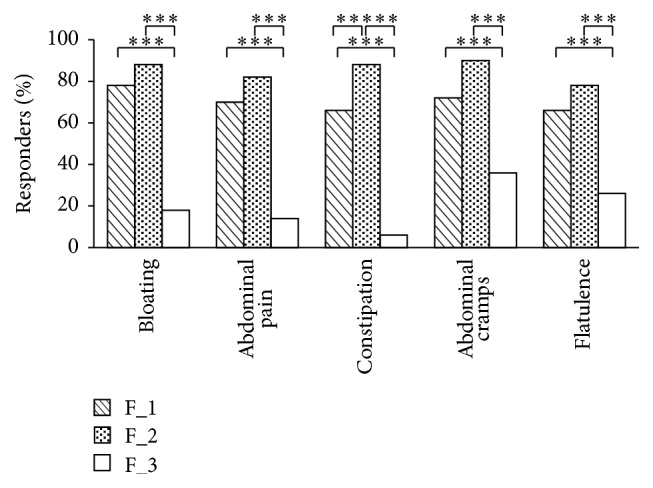
Percentage of responders to IBS-C related symptom during the treatment period (*t*60, days) with probiotic formulations F_1 and F_2. The Responders was defined as the subject reporting a decrease of symptoms of at least 30% compared to the basal condition for at least 50% of the intervention time. Bloating, abdominal pain, constipation, abdominal cramps, and flatulence symptoms were assessed on a numbering scale from 0 to 10 for each item subjects scored. Data are mean ± SE. Upon the square brackets are reported the intergroups F_1 and F_2 (versus placebo F_3) statistical analysis (^*∗∗∗*^ *P* < 0.001). The intergroups F_1 versus F_2 statistical analysis (^*∗∗*^ *P* < 0.01) is also reported.

The results of IBS-C related symptoms amelioration of the responder are reported in Figure 3. The number of responders to treatment was defined as the subject reporting a decrease of symptoms of at least 30% compared to the basal condition for at least 50% of the intervention time. Internal consistency for each item, over time, was good (Cronbach's alpha > 0.88). The percentage of responders for each clinical symptom was higher in the F_1 and F_2 group when compared to placebo F_3 (F_1 versus F_3, in the range of 66%–78% versus 6%–36%; and F_2 versus F_3 in the range of 78%–90% versus 6%–36%) (*P* < 0.001). Neither statistical nor clinically significant differences were detected between F_1 and F_2 except for constipation symptom which was less significant during the treatment (*P* < 0.01).

The results of HR-QOL are reported in Table 4. Data were reported as the sum of each score given by the subject to each item of the HR-QOL questionnaire. Internal consistency for each item, over time, was good (Cronbach's alpha > 0.86). The HR-QOL was ameliorated for subjects treated with both F_1 and F_2 during the treatment period. Relatively to baseline, the sum of each score given by the subject to each symptom during the treatment (*t*60) was significantly reduced in the probiotics group (31.2 ± 1.0 → 20.2 ± 0.9, *P* < 0.001, for F_1 group; and 32.0 ± 0.9 → 20.4 ± 0.9, *P* < 0.001, for F_2 group), but not so clinically relevant in the placebo group (30.0 ± 0.9 → 26.9 ± 1.2, *P* < 0.001, for F_3 group). As expected, mild amelioration of HR-QOL was seen in the placebo-treated subjects, probably due to the placebo effect. The intergroup amelioration of HR-QOL during the treatment (*t*60) was bigger in the actives-treated (F_1 and F_2) subjects compared to placebo-treated (F_3) subjects (F_1 versus F_3, 20.2 ± 0.9 versus 26.9 ± 1.2, and F_2 versus F_3, 20.4 ± 0.9 versus 26.9 ± 1.2, *P* < 0.001). Neither statistical nor clinically significant differences were found between F_1 and F_2.

In addition, the analysis of the stool type classification was performed (data not shown). The numbers of samples with values of 3, 4, and 5, according to Bristol scale and representing a healthy bowel movement were calculated. Significant differences were observed between F_1 or F_2 with respect to F_3, representing an increase in the number of stool samples with a healthier characteristic in the probiotic treated groups.

### 3.3. Secondary Efficacy Endpoint

**Figure 4:**
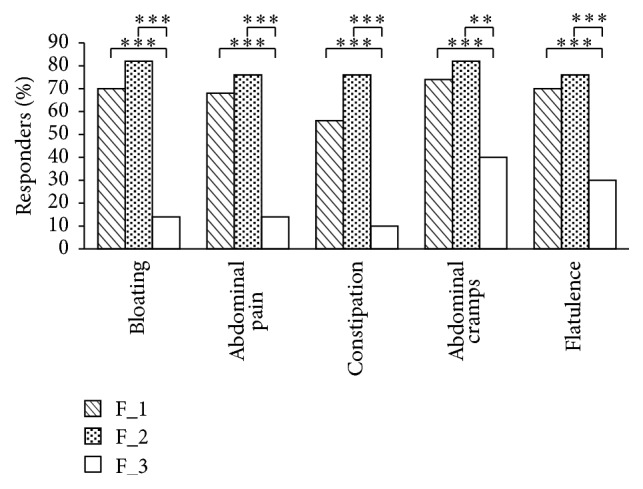
Percentage of responders to IBS-C related symptom at the follow-up period, that is, 30 days after the last product intake (*t*90, days) of probiotic formulations F_1 and F_2. The Responders was defined as the subject reporting a decrease of symptoms of at least 30% compared to the basal condition for at least 50% of the intervention time. Bloating, abdominal pain, constipation, abdominal cramps, and flatulence symptoms were assessed on a numbering scale from 0 to 10 for each item subjects scored. Data are mean ± SE. Upon the square brackets are reported the intergroups F_1 and F_2 (versus placebo F_3) statistical analysis (^*∗∗∗*^ *P* < 0.001). The intergroups F_1 versus F_2 statistical analysis (^*∗∗*^ *P* < 0.01) is also reported.

In order to assess the maintenance of the obtained effects, the study design foresaw a further 30-day follow-up period after the 60-day product intake period. The results of IBS-C related symptoms maintenance for responder are reported in Figure 4. During the follow-up period (from day 61 to day 90), the percentage of responders for each clinical symptom was higher in the F_1 and F_2 groups when compared to placebo F_3 (F_1 versus F_3, in the range of 56%–74% versus 10%–40%; and F_2 versus F_3 in the range of 76%–82% versus 10%–40%) (*P* < 0.001). Neither statistical nor clinically significant differences were detected between F_1 and F_2 except for abdominal cramps symptom which was less significant during the follow-up period (*P* < 0.01).

The results of HR-QOL are reported in Table 4. Data were reported as the sum of each score given by the subject to each item of the HR-QOL questionnaire. Data obtained 30 days (*t*90) after the last product intake were lower in the probiotic groups with respect to the basal scoring of symptoms at *t*0 (31.2 ± 1.0 → 22.2 ± 1.0, *P* < 0.001, for F_1 group; and 32.0 ± 0.9 → 22.0 ± 0.8, *P* < 0.05, for F_2 group), but not so clinically relevant in the placebo group (30.0 ± 0.9 → 28.7 ± 1.2, *P* < 0.01, for F_3 group).

The intergroup amelioration of HR-QOL was bigger in the actives-treated (F_1 and F_2) subjects compared to placebo-treated (F_3) subjects (22.2 ± 1.0 versus 28.7 ± 1.2 and 22.0 ± 0.9 versus 28.7 ± 1.2, *P* < 0.001). Neither statistical nor clinically significant differences were detected between F_1 and F_2.

Comparing follow-up period (*t*90) with the end of treatment period (*t*60), the HR-QOL was not significantly different for both F_1 and F_2 probiotic groups, indicating the maintenance of the obtained effects.

### 3.4. Fecal Microbiology Analysis by Quantitative PCR

DNA was extracted from fecal samples of the subjects enrolled in this study at the times *t*0, *t*10, *t*30, *t*60, and *t*90 from the first ingestion of the probiotic formulations. The qPCR analysis demonstrated that the species-specific sequences associated with the probiotics of the formulations were detected only in fecal DNA from subjects treated with the formulations F_1 and F_2 and not with the formulation F_3 and that no significant difference was detected between the two kinds of formulations.

**Figure 5:**
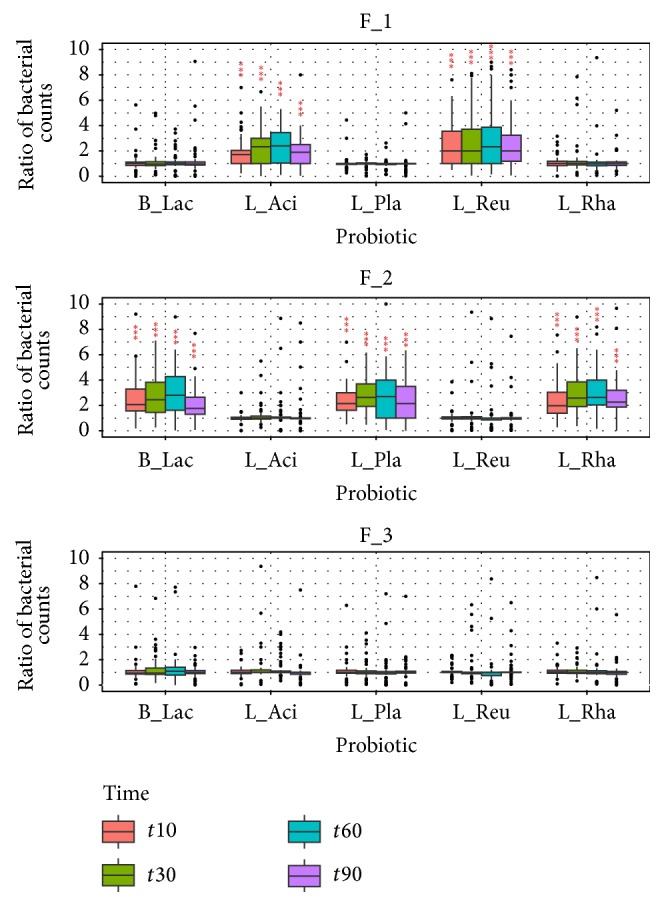
Ratio of probiotics of formulations (F_1 and F_2, versus F_3) by qPCR of species-specific sequences at the different times of treatment versus the amount at the baseline time point, expressed as bacterial counts. Upon the bars is reported the statistical analysis between treatments (^*∗∗∗*^ *P* < 0.001).

The qPCR assay for* L. plantarum *and* L. rhamnosus* (contained in the mix F_2) demonstrated a quite similar quantity of these probiotic bacteria during the times of treatment, while* B. animalis *subsp.* lactis* decreases at time 90 after the follow-up period. Concerning results of probiotics contained in the formulation F_1, we can observe that* L. acidophilus* increases during the treatment but decreases after the follow-up period, while the quantity of* L. reuteri* was quite similar during all the period of treatment including time *t*90 (Figure 5). All these results indicate that all probiotics utilized in this study were enhanced in the gut tract after their ingestion at least for 90 days; the only exception was observed for* B. animalis *subsp*. lactis* in which a lower concentration of this probiotic in the postintervention samples was obtained.

## 4. Discussion

Probiotics exert their actions through interaction with host intestinal cells. Their supplementation significantly modifies the intestinal microbiota by increasing lactobacilli and bifidobacteria that can improve through the combination with specific probiotics providing a health benefit to the host [27]. Multispecies probiotics may have a variety of different beneficial effects particularly on IBS symptoms because each species acts in a particular way on the gastrointestinal tract, and two or more species acting together may have a synergistic effect [15].

Although several trials have demonstrated the superiority of probiotics (above all, lactobacilli and bifidobacteria) over placebo in controlling IBS symptoms [16–18], however, given the controversies in IBS pathophysiology or lack of clear evidence for gut microbiota abnormalities in patients with IBS, additional randomized clinical trials with appropriate endpoints and design are needed to evaluate to which extent probiotics are a useful therapeutic strategy in the management of IBS symptoms.

In this study, a randomized, double-blind, placebo-controlled clinical trial with two formulations containing different probiotics was developed and the evaluation of gut microbiota assessment and gastrointestinal benefits of IBS-C patients was determined until 90 days. Multispecies probiotics were used for the treatment of IBS-C in our study:* L. acidophilus, L. reuteri, L. plantarum, L. rhamnosus*, and* B. animalis *subsp.* lactis*. Indeed, it is known that the level of bifidobacteria and lactobacilli species is lower in IBS patients compared to healthy persons [28, 29] and several studies show that the supplementation of them, or mixtures including species of these genera, is effective in alleviating symptoms of IBS. Moreover, the selected strains were already known for their effect on intestinal cell lines as previously reported [19].

To investigate the alterations in the intestinal microbiota, the number of lactobacilli and bifidobacteria present in fecal samples of recruited subjects was determined by quantitative real-time PCR. The numbers of* Lactobacillus* spp. and* Bifidobacterium* spp. of the mixtures (F_1 and F_2) increased during the times of treatment until 60 days in the probiotic groups, with respect to the placebo group (F_3). All the species included in the formulations remained in the gut also 30 days after the follow-up from the last ingestion, except for* Bifidobacterium*. Our results confirmed data reported by Kajander et al. These authors demonstrated that all supplemented strains remained stable during the treatment, with the exception of* Bifidobacterium* species which decreased after treatments with a multispecies probiotic mixture [14].

Moreover, in our study, significant differences in the number of responders to the severity of symptoms were recorded between the two probiotic mixtures, F_1 and F_2, with respect to placebo F_3 group (*P* < 0.001). No significant differences were registered between F_1 and F_2 (*P* > 0.05), and the effects of both of them are significant when compared to their respective baselines. These data are in agreement with previous data from multispecies probiotics treatment of IBS subjects [14, 15]. Compared with placebo, probiotic groups F_1 and F_2 were effective for the primary efficacy endpoints of the study as well as for the secondary endpoints, that is, the maintaining of the obtained effects 30 days after the last product intake. The change of symptoms is correlated to the improvement in the quality of life and resulted in being significantly higher in the probiotic groups compared with the placebo group. Although a great number of data deriving from literature [16–18, 30] indicate that probiotics may be helpful in the treatment of IBS symptoms, in particular with respect to constipation, their conclusions vary because of inadequate sample size, type of study design, and use of various probiotic strains. These data are in agreement with our data concerning the improvement of specific probiotic treatment versus placebo (specifically in patients with IBS-C) for some of the endpoints, improving symptoms such as pain, flatulence, and bloating, but not others (transit time and urgency and abdominal cramps) [30]. Moreover, in most of the reported cases, the decrease in constipation frequency score was approximately twofold greater in the probiotic groups than in the placebo groups and these results are in line with data deriving from our clinical study.

Thus, the clinical improvement of this study may be associated with the maintenance (species-specific) of the compositional stability of the intestinal microbiota from probiotics consumption and with their positive effects in subjects affected by irritable bowel syndromes.

## 5. Conclusions

In conclusion, the different species of probiotics administered to the IBS-C subjects determine a cooccurrence between the changes in the analysed probiotic groups and an improvement of IBS-C symptoms. This study represents the development of a clinical trial that can support the role of intestinal bacteria in the IBS diseases and the potential role of probiotics belonging to various species in the management of these disorders.

## Figures and Tables

**Table 1:** List of the strains used in this study, deposit number, and the most relevant antimicrobial activities described in Presti et al. 2015 [19]. Abbreviations used in the present work were also included.

Probiotic strain	Deposit number	Antimicrobial activity *vs*	Abbr.
*Lactobacillus rhamnosus* LRH020 (formerly PBS070)	DSM 25568	*C. albicans; E. faecalis; P. aeruginosa; S. aureus; E. coli*	L_Rha
*Lactobacillus plantarum *PBS067	DSM 24937	*C. albicans; E. faecalis; P. aeruginosa; S. aureus; E. coli*	L_Pla
*Bifidobacterium animalis *subsp. *lactis *BL050 (formerly PBS075)	DSM 25566	*E. faecalis; P. aeruginosa; E. coli*	B_Lac
*Lactobacillus acidophilus *PBS066	DSM 24936	*C. albicans; E. faecalis; P. aeruginosa; S. aureus; E. coli*	L_Aci
*Lactobacillus reuteri *PBS072	DSM 25175	*E. faecalis*	L_Reu

**Table 2:** List of primers used in this study.

Probiotic	Primer code	Sequence (5′ → 3′)	DNA region	Amplified length (bp)
*L. rhamnosus*	LraF	CTAGCGGGTGCGACTTTGTT	16S/23S IS	123 bp
	LraR	CAGCGGTTATGCGATGCGAA		

*L. plantarum*	Lpl2F	CATTGGAACCGAACCAGTTG	16S/23S IS	203 bp
	Lpl2R	CGGTGTTCTCGGTTTCATTATG		

*B. animalis *subsp.* lactis*	AnimF	GCACGGTTTTGTGGCTGG	pre16S	171 bp
	AnimR	GACCTGGGGGACACACTG		

*L. acidophilus*	Lacid2F	GGGCAAATCACGAACGAGTA	pre16S	132 bp
	Lacid2R	CTTTGTTTTCGTTCGCTTCA		

*L. reuteri*	Lreu2F	GTTGACGAAAGAATGAAATCCA	pre16S	118 bp
	Lreu2R	TCATGTCGTCAATCAGATGTCA		

**Table 3:** Demographic and baseline characteristics of the subjects of the clinical study^*∗*^. Data are mean ± SE.

	F_1	F_2	F_3
Number of subjects	50	50	50
Age	36.0 ± 11.9	38.0 ± 12.1	38.1 ± 13.5
Bloating (VAS)	6.3 ± 0.2	6.2 ± 0.2	6.1 ± 0.2
Abdominal pain	5.0 ± 0.2	4.9 ± 0.2	4.8 ± 0.2
Constipation	6.6 ± 0.1	6.5 ± 0.1	6.1 ± 0.2
Abdominal cramps	4.2 ± 0.2	3.9 ± 0.2	4.1 ± 0.2
Flatulence	4.6 ± 0.3	4.4 ± 0.2	4.4 ± 0.2

^*∗*^There were no statistically significant differences between the three groups.

**Table 4:** HR-QOL amelioration at the different times of treatment (*t*0, *t*10, *t*30, and *t*60, days) and at the follow-up period, that is, 30 days after the last product intake (*t*90, days) between F_1 and F_2 groups compared with F_3. Bloating, abdominal pain, constipation, abdominal cramps, and flatulence were assessed on a numbering scale from 0 to 10 for each item subjects scored. Data are mean ± SE. In brackets is reported the intergroups (versus placebo) statistical analysis.

Time (days)	F_1	*P* value	F_2	*P* value	F_3	*P* value	F_1 *vs* F_3 (*P* value)	F_2 *vs* F_3 (*P* value)
0	31.2 ± 1.0		32.0 ± 0.9		30.0 ± 0.9			
10	28.3 ± 0.9	<0.01	27.0 ± 0.8	<0.001	28.9 ± 1.0	>0.05	>0.05	>0.05
30	23.1 ± 0.9	<0.001	22.8 ± 0.8	<0.001	27.8 ± 1.1	<0.05	<0.01	<0.001
60	20.2 ± 0.9	<0.001	20.4 ± 0.9	<0.001	26.9 ± 1.2	<0.001	<0.001	<0.001
90	22.2 ± 1.0	<0.001	22.0 ± 0.8	<0.05	28.7 ± 1.2	<0.01	<0.001	<0.001
