# Corrigendum to “A Randomized, Double-Blind, Placebo-Controlled Trial: The Efficacy of Multispecies Probiotic Supplementation in Alleviating Symptoms of Irritable Bowel Syndrome Associated with Constipation”

**DOI:** 10.1155/2019/9042956

**Published:** 2019-04-09

**Authors:** Valerio Mezzasalma, Enrico Manfrini, Emanuele Ferri, Anna Sandionigi, Barbara La Ferla, Irene Schiano, Angela Michelotti, Vincenzo Nobile, Massimo Labra, Patrizia Di Gennaro

**Affiliations:** ^1^Department of Biotechnology and Biosciences, University of Milano-Bicocca, Piazza della Scienza 2, 20126 Milan, Italy; ^2^Farcoderm Srl., Via Angelini 21, San Martino Siccomario, 27028 Pavia, Italy

In the article titled “A Randomized, Double-Blind, Placebo-Controlled Trial: The Efficacy of Multispecies Probiotic Supplementation in Alleviating Symptoms of Irritable Bowel Syndrome Associated with Constipation” [1], the number of probiotics in the formulation mixtures in subsection “2.1. Study Design” in Materials and Methods was written incorrectly as follows:

“The composition of the probiotic mix F_1 was as follows: 5 × 10^9^ CFU* L. acidophilus* (30 mg as lyophilized), 5 × 10^9^ CFU* L. reuteri* (30 mg as lyophilized), 330 mg inulin, 5 mg silica, and 5 mg talc. The F_2 composition was as follows: 5 × 10^9^ CFU* L. plantarum* (12 mg as lyophilized), 5 × 10^9^ CFU* L. rhamnosus* (20 mg as lyophilized), 5 × 10^9^ CFU* B. animalis* subsp.* lactis* (60 mg as lyophilized), 298 mg inulin, 5 mg silica, and 5 mg talc. Placebo (F_3) composition was as follows: 390 mg inulin, 5 mg silica, 5 mg talc.”

It should be corrected to the following: “The composition of the probiotic mix F_1 was as follows: 2 × 10^9^ CFU* L. acidophilus* (30 mg as lyophilized), 2 × 10^9^ CFU* L. reuteri* (30 mg as lyophilized), 330 mg inulin, 5 mg silica, and 5 mg talc. The F_2 composition was as follows: 2 × 10^9^ CFU* L. plantarum* (12 mg as lyophilized), 2 × 10^9^ CFU* L. rhamnosus* (20 mg as lyophilized), 2 × 10^9^ CFU* B. animalis* subsp.* lactis* (60 mg as lyophilized), 298 mg inulin, 5 mg silica, and 5 mg talc. Placebo (F_3) composition was as follows: 390 mg inulin, 5 mg silica, and 5 mg talc.”

## References

[1] Mezzasalma V., Manfrini E., Ferri E. (2016). A randomized, double-blind, placebo-controlled trial: the efficacy of multispecies probiotic supplementation in alleviating symptoms of irritable bowel syndrome associated with constipation. *BioMed Research International*.

